# Neutrophil‐to‐lymphocyte ratio and D‐dimer are biomarkers of death risk in severe COVID‐19: A retrospective observational study

**DOI:** 10.1002/hsr2.514

**Published:** 2022-02-09

**Authors:** Patrícia Oliveira Cunha Terra, Camila Dermínio Donadel, Luciana Correa Oliveira, Mayra Gonçalves Menegueti, Maria Auxiliadora‐Martins, Rodrigo Tocantins Calado, Gil Cunha De Santis

**Affiliations:** ^1^ Department of Medical Imaging, Hematology, and Oncology Ribeirão Preto Medical School, University of São Paulo São Paulo Brazil; ^2^ Regional Blood Center of Ribeirão Preto Ribeirão Preto Medical School, University of São Paulo São Paulo Brazil; ^3^ Ribeirão Preto Nursing School University of São Paulo São Paulo Brazil; ^4^ Division of Intensive Medicine Ribeirão Preto Medical School, University of São Paulo São Paulo Brazil

**Keywords:** biomarkers, COVID‐19, critical care, D‐dimer, neutrophil‐to‐lymphocyte ratio

## Abstract

**Background and Aims:**

Over 4 million deaths from coronavirus disease (COVID)‐19 have been reported in the world. Several biomarkers have been identified that predict disease severity, but there is still a need to identify biomarkers for death risk in severe COVID‐19. We aim to define amongst the biomarkers already identified those which are mostly associated with increased death rate in patients with severe COVID‐19.

**Methods:**

In this retrospective study conducted in three public hospitals linked to the Medical School of Ribeirão Preto, Brazil, patients with severe COVID‐19 were evaluated regarding biomarkers (neutrophil‐to‐lymphocyte ratio‐NLR, D‐dimer, fibrinogen) of death risk, obtained before administration of corticosteroids.

**Results:**

Thirty‐nine (32.8%) of the 119 patients included (104 [87.4%] on mechanical ventilation) died during hospitalization. Non‐survivor group had higher median (range) NLR (12.63 [2.6‐115] vs 7.43 [0.43‐31.8]; *P* = .001), D‐dimer (2.17 [0.27‐20.00] vs 1.57 [0.28‐20.00]; *P* = .03), but lower fibrinogen (631 [353‐1078] vs 705 [407‐1200]; *P* = .02). The group with NLR ≥ 10 and D‐dimer ≥ 2 μg/mL had a higher death risk than the group with NLR < 10 and D‐dimer < 2 μg/mL (OR: 5.39; CI 95%: 1.5‐19.42; *P* = .01).

**Conclusion:**

High NLR and D‐dimer, especially when combined, are predictors of death risk for patients with severe COVID‐19 and should be incorporated into their evaluation.

## 1 INTRODUCTION

At the end of 2019 a novel coronavirus, known as SARS‐CoV‐2, was identified in Wuhan, China.^1^ Since then, globally, over 200 million confirmed cases of coronavirus disease 2019 (COVID‐19) have been reported. In Brazil, since February 2020, more than 20 million people have been diagnosed with COVID‐19, of which more than 570 000 died of this disease.

The clinical picture of COVID‐19 is characterized by fever, dry cough, dyspnea, fatigue, myalgia, anosmia, and ageusia.^2^ Findings in chest radiography or computerized tomography imaging usually show bilateral pulmonary ground‐glass opacifications, mainly in posterior and peripheral areas of the lungs.^3^ Mortality rate in COVID‐19 has been reported to be somewhat less than 1%.^4^ The mortality predictors initially identified were older age, elevation of the D‐dimer above 1 μg/L and higher SOFA score.^5^ Other factors were associated with increased risk of death, for instance, male gender, obesity, cardiovascular disease, diabetes respiratory disease, and ABO blood type.^6^, ^7^, ^8^

Several biomarkers have been identified in order to predict the severity of COVID‐19, such as lymphocyte count, neutrophil‐to‐lymphocyte ratio (NLR), C‐reactive protein (CRP), interleukin‐6 (IL‐6), and D‐dimer. NLR, CRP, and IL‐6 reflect inflammation status, whereas D‐dimer the coagulation, the two main psychopathological aspects of COVID‐19.^9^, ^10^, ^11^, ^12^

In this study, we aim to define amongst the biomarkers already identified those which are mostly associated with increased death rate in patients with severe COVID‐19 admitted to intensive care unit (ICU).

## 2 MATERIALS AND METHODS

### 2.1 Study design and participants

This is a retrospective observational study conducted at three hospitals linked to the Medical School of Ribeirão Preto of the University of São Paulo. This study included patients ≥18 years of age with severe COVID‐19 admitted to ICU from April 2020 to November 2020. Exclusion criteria were: previous severe comorbidities, such as cancer, cirrhosis, HIV infection, chronic renal failure, cardiac failure and others associated with diminished life expectancy. Also, patients using corticosteroids before ICU admission were excluded.

The disease severity was defined according to the WHO Working Group on the Clinical Characterization and Management of COVID‐19 infection classification. Moreover, we applied Simplified Acute Physiologic Score 3 (SAPS‐3), which is a score system employed to predict death risk on admission to ICU. A higher score indicates higher risk of death.^13^, ^14^

### 2.2 Data collection

Epidemiological, demographic, clinical, laboratory, treatment, and outcome data were extracted from electronic medical records using a standardized data collection form. All data were checked by two physicians.

### 2.3 Laboratory procedures

The method for laboratory confirmation of COVID‐19 was the SARS‐CoV‐2 detection in respiratory specimens by real‐time polymerase chain reaction. Routine blood tests were: complete blood count, coagulation profile (including D‐dimer and fibrinogen), serum biochemical tests, serum ferritin, lactate dehydrogenase (LDH), homocysteine and C‐reactive protein (CRP). The results analyzed were obtained between the date of admission to ICU and before administration of corticosteroids.

### 2.4 Statistical analysis

Continuous variables were expressed as means and SD or medians and range, according to distribution characteristics (the Kolmogorov‐Smirnov test was employed to define distribution characteristics). Categorical variables were summarized as the counts and percentages in each category. To compare quantitative variables between the two groups a Student *t* test was used if the variables had Gaussian distribution, and a Mann‐Whitney *U* test if they did not. Statistical comparison between groups for categorical variables were performed by employing chi‐square or Fisher's exact test. After univariate analysis, we performed multivariate analysis including only the comparisons with *P* < .20. The results were considered to be statistically different when the *P*‐value was below .05 (by two‐tailed testing). Statistical analyses were performed using statistical GraphPAD Prism Software, version 9.1 (GraphPad software, Inc., San Diego, CA, USA) and Stata for Windows, version 14 (StataCorp, College Station, Texas, USA).

## 3 RESULTS

**FIGURE 1:**
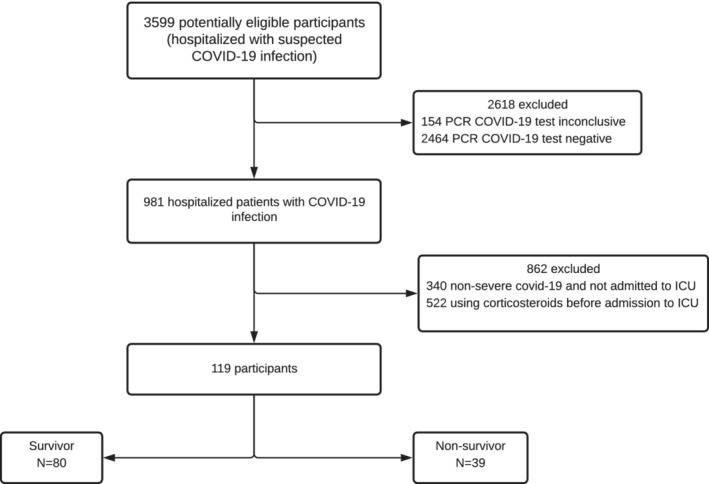
Flow diagram of participants into the study. DD, D‐dimer; ICU, intensive care unit; NLR, neutrophil‐to‐lymphocyte ratio; PCR, real‐time polymerase chain reaction

A total of 119 patients, selected among 3599 hospitalized because of suspected COVID‐19, fulfilled inclusion criteria (Figure 1) and were enrolled in this study at three connected centers in the state of São Paulo (Brazil), from April 2020 to November 2020.

Invasive mechanical ventilation was required in 104 (87.4%) patients at some moment during in‐hospital care and 39 (32.8%) of the 119 patients died during hospitalization. All of them were treated with corticosteroids (methyl‐prednisolone or dexamethasone) during their stay in ICU. Furthermore, 87 of the patients were included in a clinical trial of convalescent plasma (CP) transfusion, 28 (32.18%) of which received CP. There was no statistical difference (*P* = .62) regarding the use of convalescent plasma between the survivors and non‐survivors (data not shown).

Patients' characteristics at ICU admission are detailed in Table 1. Non‐survivor group was older and had a higher rate of blood hypertension and a higher value of SAPS‐3 score. The non‐survivor group also presented a higher neutrophil‐to‐lymphocyte ratio (NLR) and D‐dimer, but a lower fibrinogen concentration (Table 2).

**TABLE 1:** Baseline demographics and clinical characteristics of study groups

	Survivor (80)	Non‐survivor (39)	*P* value
Age (yr), mean ± SD	56.7 ± 12.3	67.3 ± 11.8	<.001
Male (%)	54 (67.5)	21 (53.9)	.16
BMI (kg/m^2^), median (range)	30.6 (20.9‐74.2)	28.9 (20.8‐52.8)	.40
Comorbidities			
Hypertension (%)	44 (55)	33 (84.6)	.002
Diabetes mellitus (%)	33 (41.3)	19 (48.7)	.56
SAPS‐3 score, mean ± SD	53.6 ± 20.8	66.4 ± 23.8	.006

*Note*: SAPS‐3 scores were assessed on admission to the study.

Abbreviations: BMI, body mass index; SAPS‐3 score, simplified acute physiology score 3.

**TABLE 2:** Biomarkers according to the two groups

	Survivor (80)	Non‐survivor (39)	*P* value
Hemoglobin (female: 12.4‐16.1 g/dL; male: 13.9‐17.7 g/dL)	12.78 ± 2.17	12.51 ± 1.91	.52
Neutrophil (*N*: 1.7‐7.2 × 10^3^/μL)	7.2 (1.9‐16.9)	10.0 (1.3‐16.2)	.052
Lymphocyte (*N*: 1.07‐3.12 × 10^3^/μL)	0.9 (0.2‐9.7)	0.8 (0.1‐2.6)	.13
NLR^a^	7.43 (0.43‐31.8)	12.63 (2.6‐115)	.001
Platelet (*N*: 166‐389 × 10^3^/μL)	246.10 ± 88.11	253.50 ± 99.05	.68
Fibrinogen (*N*: 200‐393 mg/dL)	705 (407‐1200)	631 (353‐1078)	.02
D‐dimer (*N*: ≤0.5 μg/mL)	1.57 (0.28‐20.00)	2.17 (0.27‐20.00)	.03
C‐reactive protein (*N*: <1.0 mg/dL)	16.18 ± 9.39	16.45 ± 9.26	.88
Ferritin (*N*: 22‐322 ng/mL)	1264 (116.9‐4368)	1073 (71.8‐8250)	.17
LDH (*N*: 120‐246 U/L)	615.9 (129‐2944)	647 (199‐4363)	.51
Homocysteine (*N*: 3.7‐13.9 μmol/L)	12.37 (2.1‐36.25)	13.02 (1.34‐24.96)	.83

*Note*: Results given as median (range) (Mann–Whitney U test) or mean ± SD (Student *t* test).

Abbreviations: LDH, lactate dehydrogenase; *N*, normal reference laboratory value; NLR, neutrophil‐to‐lymphocyte ratio.

There is no consensual normal reference value.

In another type of analysis, we divided the participants into two groups according to the NLR cutoff of 10 (median of all patients was 9.29). Similarly, we divided the participants into two groups according to the D‐dimer cutoff of 2 (median of all patients was 1.89 μg/mL). When we combined the two biomarkers and divided the participants into two groups (NLR ≥ 10 and D‐dimer ≥ 2 μg/mL vs NLR < 10 and D‐dimer < 2 μg/mL) it was observed that the first group had a higher death risk than the second (relative risk: 2.714; CI 95%: 1.516‐4.998) (Table 3).

**TABLE 3:** Death rate according to combined NLR and D‐dimer

	NLR ≥ 10/DD ≥ 2 (25)	NLR < 10/DD < 2 (34)	RR (95% CI)	*P* value
Survivor	10	28		
Non‐survivor (%)	15 (60)	6 (17.65)	2.714 (1.516–4.998)	.001

Abbreviations: CI, confidence interval; DD, D‐Dimer (μg/mL); NLR, neutrophil‐to‐lymphocyte ratio; RR, relative risk.

In the multivariate analysis, it was observed that NLR ≥ 10 and D‐dimer ≥ 2 μg/mL were associated with a higher death rate (Table 4).

**TABLE 4:** Results of a multivariate logistic regression model to predict death risk in COVID‐19

Variables	Odds ratio (95% CI)	*P* value
Age (year)	1.025 (0.97‐1.08)	.34
SAPS‐3 score	1.020 (0.99‐1.05)	.15
Hypertension	2.687 (0.66‐10.93)	.17
Fibrinogen	0.996 (0.992‐0.999)	.02
NLR ≥ 10/DD ≥ 2	5.39 (1.5‐19.42)	.01

Abbreviations: CI, confidence interval; DD, D‐Dimer; NLR, neutrophil‐to‐lymphocyte ratio.

## 4 DISCUSSION

This retrospective observational study identified perhaps the two most important biomarkers for death risk in patients with severe COVID‐19, which are neutrophil‐to‐lymphocyte ratio and D‐dimer. It is important to emphasize that the patients had an extremely severe clinical picture, as approximately 90% of them were on mechanical ventilation and one third died in hospital.

The differences in epidemiological and clinical characteristics between the groups of survivors and non‐survivors have already been described in other studies, which identified factors and biomarkers associated with increased death risk.^5^, ^8^ In our study, the clinical factors associated with higher mortality, as expected, were older age, blood hypertension and higher value of SAPS‐3. As for laboratory evaluation, higher NLR and D‐dimer and lower fibrinogen levels on admission were associated with an increased risk of death.

NLR is a marker previously used as a prognostic factor for patients with sepsis, as it is an early indicator of increased systemic inflammation.^10^, ^15^ Previous studies reported that increased NLR is found in patients with severe COVID‐19 compared to those with non‐severe disease, and possibly reflects an immune system dysregulation.^15^, ^16^, ^17^, ^18^, ^19^ Jimeno et al observed that patients with severe clinical courses presented higher NLR at admission and higher NLR peak than that observed in non‐severe group, and hypothesized that NLR is a marker of endothelial dysfunction.^20^ In our study, we identified that NLR is not only important to stratify the severity of the disease, but also to predict mortality in severe cases.

Elevation of D‐dimer indicates the activation of coagulation and fibrinolysis and occurs in conditions such as thrombosis, pregnancy, cancer, and inflammation. The COVID‐19 disease promotes a hypercoagulable state caused by the imbalance between the pro‐ and anti‐inflammatory response, endothelial dysfunction with excess thrombin generation, hypoxia and immobilization.^21^, ^22^ Increased level of D‐dimer was suggested as a good predictor of mortality in COVID‐19, especially if the value was higher than 2 μg/mL on admission to hospital. In our study, D‐dimer ≥ 2 μg/mL denoted a higher risk of death in severe COVID‐19.

Increased fibrinogen concentrations have been described in critically ill patients, including patients with COVID‐19.^23^, ^24^ Tang et al showed that even though patients hospitalized for COVID‐19 presented elevated fibrinogen concentration on admission, after 10 to 14 days the non‐survivor group had lower fibrinogen levels.^25^ We found in our study increased fibrinogen levels compatible with severe COVID‐19, however, in the non‐survivor group fibrinogen values were lower than those found in the survivor group. We hypothesized that in the non‐survivor group a progression to a later stage of disseminated intravascular coagulation may be already occurring, with increased consumption of fibrinogen, however, more studies are necessary to investigate this issue.

Hemoglobin concentration was similar in both groups and was within the reference values on admission to ICU. We believe that this finding may be explained by the fact that the decrease of hemoglobin concentration is a later event in an inflammatory picture and our patients here were evaluated relatively early, before a clear impact of inflammation on erythropoiesis.^26^

It is important to highlight that patients with NLR ≥ 10 and D‐dimer ≥ 2 μg/mL had a significantly higher death risk than the group with values below those defined above. Both parameters combined represent a strong prognostic factor to be considered on admission of patients with severe COVID‐19. What is more, beyond their statistical significance, they portray the two most important aspects of COVID‐19 pathophysiology: inflammation and coagulation abnormalities.

Fu et al reported that NLR and D‐dimer levels were higher in patients with severe COVID‐19 compared with the mild/moderate group on days 1, 7, and 14 of the disease.^27^ Ye et al found that in hospitalized patients initial and peak D‐dimer and NLR were higher in intubated and in deceased patients.^28^ Our study adds to the data that combined NLR and D‐dimer may not only aid in the differentiation between severe and non‐severe COVID‐19 as described in the literature, but also predict the death risk in those patients with severe COVID‐19.

The main limitation of our study is its retrospective design, carried out in a single region of Brazil. The sample size could be considered relatively small, however, it had a sufficient size to enable us to obtain significant results. We believe that a strong point of our study is that the biomarkers of death risk were evaluated in patients with severe COVID‐19, all of them admitted to ICU. D‐dimer and, especially, NLR are universally available exams, including in small units of care in low‐income countries. Another strong point of this study is that the participants were recruited from a miscegenated population, which perhaps confers to our findings a universal validity. It is important to emphasize that the NLR should be obtained before introduction of corticosteroids, which knowingly increases neutrophil and decreases lymphocyte counts.

## 5 CONCLUSIONS

High NLR (≥10) and D‐dimer (≥2.0 μg/mL), especially when combined, are strong predictors of death risk for patients with severe COVID‐19. These tests should be incorporated into the general evaluation of death risk for patients with severe COVID‐19 because of their clinical significance.

## FUNDING

Fundação de Amparo à Pesquisa do Estado de São Paulo (FAPESP) for the funding of this research (Grant No. 2020/05367‐3). FAPESP had no interference in the study design, which was the authors exclusive responsibility.

## CONFLICT OF INTEREST

The authors have no conflict of interest to disclose.

## TRANSPARENCY STATEMENT

Patrícia Oliveira da Cunha Terra affirms that this manuscript is an honest, accurate, and transparent account of the study being reported; that no important aspects of the study have been omitted; and that any discrepancies from the study as planned (and, if relevant, registered) have been explained.

## AUTHORS CONTRIBUTIONS

Conceptualization: Gil Cunha De Santis.

Data Curation: Patrícia Oliveira da Cunha Terra, Camila Dermínio Donadel, Mayra Gonçalves Meneguetti, Gil Cunha De Santis.

Formal analysis: Patrícia Oliveira da Cunha Terra, Camila Dermínio Donadel, Mayra Gonçalves Meneguetti, Gil Cunha De Santis.

Investigation: Patrícia Oliveira da Cunha Terra, Camila Dermínio Donadel, Gil Cunha De Santis.

Writing – Original Draft: Patrícia Oliveira da Cunha Terra, Camila Dermínio Donadel, Gil Cunha De Santis.

Writing – Review and Editing: Patrícia Oliveira da Cunha Terra, Camila Dermínio Donadel, Gil Cunha De Santis.

Supervision: Luciana Correa Oliveira, Maria Auxiliadora‐Martins, Rodrigo Tocantins Calado.

All authors have read and approved the final version of the manuscript.

Patrícia Oliveira da Cunha Terra had full access to all of the data in this study and takes complete responsibility for the integrity of the data and the accuracy of the data analysis.

## ETHICS STATEMENT

This study was approved by the institutional review board (Comitê de Ética em Pesquisa do Hospital das Clínicas de Ribeirão Preto; CAAE 50193221.7.0000.5440), and was performed in accordance to the Helsinki Declaration of 1964 and its later amendments. As this is a retrospective study, the ethical review board agreed to waiver the signed consent by the patients.

## Data Availability

The authors confirm that the data supporting the findings of this study are available within the article.
